# Development of a Nano-Real-Time Polymerase Chain Reaction (RT-PCR) Kit for Detection and Genotyping of High-Risk Human Papillomavirus (HPV) Strains Using Dedicated TaqMan Probes

**DOI:** 10.7759/cureus.100905

**Published:** 2026-01-06

**Authors:** Mohammad Panji, Mohammad Hossein Modarresi, Zahra Azizi, Moloud Absalan, Elahe Motevaseli

**Affiliations:** 1 Department of Molecular Medicine, School of Advanced Technologies in Medicine, Tehran University of Medical Sciences, Tehran, IRN; 2 Department of Medical Genetics, School of Medicine, Tehran University of Medical Sciences, Tehran, IRN

**Keywords:** gold nanoparticles (aunps), hpv types, molecular diagnosis of infectious diseases, nano-real time pcr, taqman probes

## Abstract

Background: Gold nanoparticles (AuNPs) have demonstrated promise in enhancing polymerase chain reaction (PCR) efficiency, leading to more precise viral detection. The integration of AuNPs into PCR protocols has been shown to improve amplification efficiency and detection specificity, thereby enabling more accurate viral diagnostics. This study developed and optimized a nano-real-time PCR (RT-PCR) assay incorporating AuNPs for human papillomavirus (HPV) detection and genotyping.

Methods: The synthesized pegylated AuNPs were characterized using ultraviolet-visible spectroscopy, dynamic light scattering (DLS), transmission electron microscopy (TEM), zeta potential analysis, and Fourier-transform infrared spectroscopy (FTIR). Different concentrations of AuNPs were tested to determine the optimal conditions of RT-PCR. The specificity and sensitivity of the assay were evaluated by testing various HPV genotypes and comparing the performance with commercial PCR kits.

Results: The results confirmed that the optimal concentration of AuNPs for improved PCR performance was 1 nM, ensuring stable amplification efficiency across different fluorophore-labeled TaqMan probes (FAM, HEX, ROX, and Cy5). The assay successfully detected HPV DNA at concentrations as low as 0.1 ng and demonstrated 100% specificity, with no cross-reactivity observed with herpes simplex virus (HSV). The detection limit of the assay was 16 copies per reaction, surpassing conventional PCR methods. Additionally, the designed kit showed comparable accuracy to commercial detection kits, further supporting its diagnostic reliability.

Conclusion: Incorporating AuNPs in RT-PCR significantly improved fluorescence signal detection, primer binding efficiency, and polymerase activity, enhancing sensitivity and specificity. Given its high diagnostic accuracy, this assay represents a promising tool for the early and reliable detection of HPV, with potential applications in clinical diagnostics and epidemiological surveillance.

## Introduction

Human papillomavirus (HPV) is a widespread virus affecting epithelial cells, and certain types, particularly high-risk types, are linked to the onset of several cancers, including cervical, anal, and head and neck cancers [1,2]. Among these, HPV types 16 and 18 are recognized for their significant potential to cause cancer [3]. Consequently, early detection and accurate typing of high-risk HPV strains in clinical settings are crucial for recognizing individuals susceptible to malignancies and guiding treatments [4,5]. At present, screening techniques like Pap smears and HPV DNA tests are commonly employed. Still, these approaches often have limitations in sensitivity, precision, and the capability to identify various HPV types correctly. To overcome these challenges, there is a need for advanced and reliable diagnostic tools that provide higher sensitivity and accuracy for HPV typing [6,7].

Polymerase chain reaction (PCR) is a widely recognized molecular method that amplifies targeted nucleic acid sequences and allows the detection of even small amounts of viral nucleic acids [8,9]. Among PCR techniques, real-time PCR (RT-PCR) has become the gold standard due to its ability to simultaneously amplify and monitor the amplification of DNA in real time, delivering quantitative information essential for evaluating viral load [10]. The creation of targeted TaqMan probes for RT-PCR provides an extra level of precision, enabling more accurate identification and quantification of specific HPV types. These probes are designed to bind to unique sequences in the viral genome, thus identifying only the intended HPV types and reducing the chance of cross-reactions with non-target types [11,12].

The incorporation of gold nanoparticles (AuNPs) into RT-PCR represents a significant advancement. AuNPs have unique optical and electronic characteristics that can improve PCR efficiency. When added to PCR, they can enhance DNA amplification efficiency by improving heat transfer, thereby accelerating reaction kinetics. Moreover, AuNPs can stabilize the reaction, reducing the potential for non-specific binding, and improve assay reliability [13-15]. Adding these nanoparticles enhances sensitivity, enabling the identification of minimal quantities of viral DNA, boosting assay specificity, and minimizing false positives and negatives, although it could impose a financial burden on users. This development can enhance the reliability and efficiency of RT-PCR, delivering quicker and more accurate diagnostic results [13,15,16].

This article reports on the design, optimization, and validation of a nano-RT-PCR kit, including a detailed evaluation of the primers and probes used. In addition to HPV detection, this technology permits precise genotyping and offers comprehensive details regarding the viral strains found in clinical samples. Genotyping of HPV enables more precise risk stratification for cancer progression, which informs clinical decision-making and improves patient management.

## Materials and methods

Chemicals and reagents

Liquid base medium, NaAuCl₄, sodium citrate, thiol-PEG3k-carboxylate, phosphate-buffered saline (PBS), and polyethylene glycol (PEG) were all purchased from Sigma-Aldrich (Germany).

Clinical sample collection

The code of ethics for this study was obtained from Tehran University of Medical Sciences (IR.TUMS.TIPS.REC.1402.195). One hundred HPV-positive clinical samples were collected from gynecologists in Valiasr Hospital (Tehran, Iran) to validate the HPV diagnostic test. The authoritative blinded histopathologists approved the presence of HPV and genotyping of the subtypes. The sampling process was performed after obtaining written informed consent from the patients and in accordance with the standard operating procedure (SOP). Sampling was performed by a gynecologist using standard tools such as a cytobrush (Arian Teb, Iran). The isolated cells were immediately transferred to liquid base medium to provide suitable conditions for maintaining the quality of the samples. After collection, the samples were stored in a freezer at -20°C.

Probe and primer design

Probes and primers were designed using Oligo 7 software to specifically bind to target sequences in the E6 and E7 genes of various HPV types (Table 1). A fluorescent reporter dye was used at the 5' end, and a quencher was used at the 3' end, as represented in Table 2. BLAST was used to check for overlap of primers and probes with other genomic sequences.

**Table 1:** Probes and primers sequences HPV, human papillomavirus; H-GPDH, human glyceraldehyde-3-phosphate dehydrogenase

HPV type	F primers	R primers	Sense	Antisense	Product length
HPV 11	CGCCATTAAACTTACAACACAG	CTTCCACTTCAGAATAGCCA	AAACACGGGAATTAACGGACAG		98
HPV 33	TTTAGGGTCCGTTTACCAG	CACTTATGCCAACGCCTA		ACACATGCCCATACCAATCG	139
HPV 35	ACAAATCACAAACGACCT	TGTACTACAACTACCACACCG		CCCCTCTGTCAACACTGTCC	147
HPV 39	ATGTTACGAGCAATTAGGAG	CACTTACAACACGAACACT	ATGAAATAGATGAACCCGACCA		132
HPV 45	TTCCCGATTATTAACTGTAGGC	AATTTATTAGGATCGGGTAAAGC	ATCCGCATATCAGTATAGGGTGTT		135
HPV 51	CGCGTTATCCACTACTACAAC	AGTAAGTCGCTGTCGTTT	CAACGACCAATCCCCTTACCAC		121
HPV 52	GGACAAGTAGATTACTATGGGTT	TGCAGGACAAACAATTACCTGA		CCCACATGTACTTCCCATACTCC	144
HPV 56	GGACAAGTAGATTACTATGGGTT	CCATGTGCTATTAGATGAAATCG	AAGGTGCTACAGATGTCAAAGTCC		157
HPV 58	TATGTTCCAGGACGCAGA	TTTTGCATTCAACGCATT	AAACCACGGACATTGCAT		104
HPV 59	AAACCAGTAACCTGCGAT	TGTTGCATTTTCATCCTCGTC		CTTCCCCATCTGTACCTTCCGA	139
HPV 6	GAAAGTGAAATAAGTCCACGATT	CTTCCACTTCAGAATAGCCA		ATCCACTGTCCGTTAGTTCCC	124
HPV 66	TATTCAGTGTATGGGGCAAC	CTTTTATGTTCACAGTGCAA	CAATAAGGTGCTACCGATGTCA		128
HPV 68	ACATTTACCTCCCGTTCC	GACGTTGCTGGTAACACA	CCTTCATTAGCGTCTACAGCATC		143
HPV 16	GATTGTCCACCATTAGAGT	CTTCACTTTTGTTAGCCTGT		ACCAAAGCCAGTATCAACCA	109
HPV 18	CCTTCTATGTCACGAGCAA	TTCTGGCTTCACACTTACAACA	ACAACAGCAGTGTAGACGGTA		143
HPV 31	CATATAGGTATTACACCGTT	TTCGGTTCACCAATTTCG		CACCAATTTCGGTTACTCC	116
H-GPDH	CTGACTTCAACAGCGACACCCA	CCACCCTGTTGCTGTAGCCA		CCAGCCCCAGCGTCAAAGGT	123

**Table 2:** Fluorescent reporter dyes for each HPV type HPV, human papillomavirus

FAM	Mix 1 with HPV 16
	Mix 2 with HPV 39
	Mix 3 with HPV 33
	Mix 4 with HPV 58
ROX	Mix 1 with HPV 18
	Mix 2 with HPV 59
	Mix 3 with HPV 68
	Mix 4 with HPV 66
Cy5	Mix 1 control
	Mix 2 control
	Mix 3 with HPV 56
	Mix 4 with HPV 51
HEX	Mix 1 with HPV 31
	Mix 2 with HPV 45
	Mix 3 with HPV 35
	Mix 4 with HPV 52

AuNPs synthesis

A 420 μL volume of a 0.125 mol/L NaAuCl₄ solution was added to 94.6 mL of stirring deionized water heated to 90°C. This was immediately followed by the rapid addition of 5 mL of a 10 mg/mL sodium citrate solution to facilitate the reduction of gold ions. The solution was heated and stirred for 20 minutes, with the progression of the reaction monitored by a color change from light yellow to gray and ultimately to a deep red, confirming the formation of AuNPs. The final suspension was allowed to cool to ambient temperature and stored at 4°C for preservation.

PEGylation of AU nanoparticles

Thiol-PEG3k-carboxylate (10 nM) was added to 500 μL of AuNPs. The mixture was then incubated for eight hours at 4°C to activate the reaction. The resulting thiol-PEG-carboxylate-modified AuNPs were then purified through two centrifugation cycles at 8000 g for 40 minutes at 4°C to remove unconjugated thiol-PEG-carboxylate. Finally, the AuNPs were resuspended with PBS after centrifugation and stored at 4°C for further use.

Characterization of AuNPs

The morphological characteristics of the synthesized AuNPs were analyzed using transmission electron microscopy (TEM) on a Thermo Fisher Scientific microscope (Themis™ ETEM, USA). Zeta potential and dynamic light scattering (DLS) measurements, which provided data on particle surface charge and hydrodynamic size, respectively, were performed on a Malvern ZS90-2027 Zetasizer Nano system.

Ultraviolet-visible (UV-vis) spectra were recorded with a Lambda 35 UV-vis spectrophotometer (PerkinElmer, USA) to monitor spectral changes in the AuNPs. Additionally, a PerkinElmer Fourier-transform infrared spectroscopy (FTIR) spectrometer was utilized to analyze the chemical bonds and functional groups on the nanoparticle surfaces.

Quantitative RT-PCR and evaluation of sensitivity and specificity

To optimize the AuNP concentration in the RT-PCR reaction, AuNPs, standard RT-PCR reagents (PCR premix, primers, and specific HPV probes), HPV viral DNA (positive sample), and negative control DNA (without HPV) were prepared in a final volume of 20 μL. For the preparation of the AuNP solution, AuNPs with different concentrations (0.5 nM, 1 nM, 1.5 nM, 2 nM) were prepared. RT-PCR reactions were performed using a Corbett Research Ltd. Rotor-Gene™ machine at each AuNP concentration to evaluate the effect of nanoparticles on amplification efficiency.

A serial dilution of viral copy numbers was created to assess the test's sensitivity. Four serial dilutions were prepared, varying from one copy to 10⁶ copies of HPV 16. The minimal detectable copy number and the sensitivity of nano-RT-PCR were compared with the commercial DynaBio™ HPV 16 & 18 Detection kit. A standard curve was then constructed. To evaluate the specificity of the test and confirm that the designed probes exclusively attach to the target virus, a concurrent analysis of two distinct viruses (HPV and herpes simplex virus (HSV)) was performed using a combined nano-RT-PCR method. To evaluate the specificity, different concentrations of HPV and HSV were prepared and used in the assay, as shown in Table 3. Master mixes were prepared by combining different concentrations of virus with appropriate reagents, and the reaction was performed using the nano-RT-PCR kit with different reaction system colors. The assay was evaluated for its ability to detect four distinct HPV genotypes: HPV 16, HPV 18, HPV 56, and HPV 31. The successful detection of all four types was achieved using master mixtures containing AuNPs.

**Table 3:** Different concentrations of HPV and HSV virus HPV, human papillomavirus; HSV, herpes simplex virus

1	HPV=1 ng
2	HPV=0.5 ng, HSV=0.5 ng
3	HPV=0.3 ng, HSV=0.7 ng
4	HPV=0.1 ng, HSV=0.9 ng
5	HSV=1 ng

The following formulas were used to evaluate the sensitivity and specificity of the test: sensitivity % = (true positives / (true positives + false negatives)) * 100; specificity % = (true negatives / (false positives + true negatives)) * 100. Furthermore, to validate the assay's specificity, sensitivity, and limit of detection (LOD), the results were benchmarked against established commercial kits, including the GENEMARKER (Germany) and IONTEK (Turkey) systems. In this article, established brand kits were utilized due to their licensing and high sensitivity and specificity. Consequently, they serve as established references for a comparative analysis of our developed kit's performance.

Statistical analysis

In this study, statistical analysis of data was performed using the one-way ANOVA test in GraphPad Prism 8 (GraphPad Software, San Diego, CA, USA). A significance level of p<0.05 was considered, and the results were reported as mean±SD.

## Results

Characterization of AuNPs

**Figure 1:**
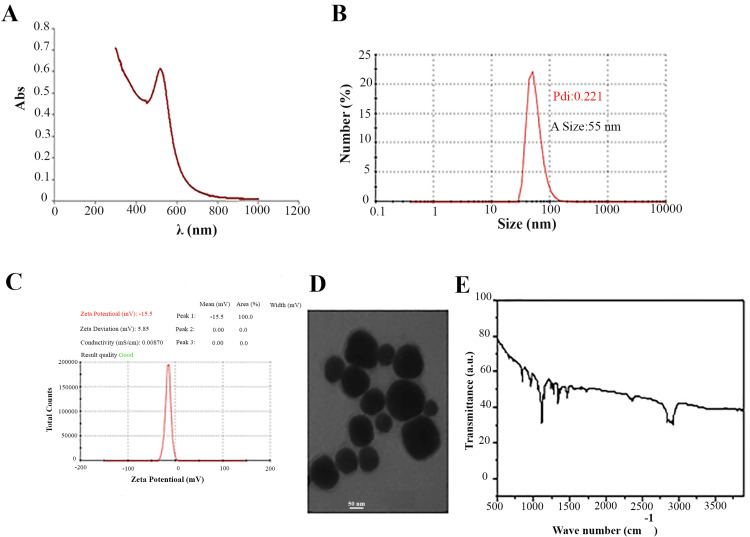
Characterization of Au nanoparticles A) UV-vis spectroscopy. B) DLS analysis results. C) Zeta potential analysis results.  D) TEM images. E) FTIR spectrum of PEG-modified AuNPs. UV-vis, ultraviolet-visible; DLS, dynamic light scattering; TEM, transmission electron microscopy; FTIR, Fourier-transform infrared spectroscopy; PEG, polyethylene glycol; AuNPs, gold nanoparticles

UV-vis spectroscopy indicated that the maximum absorption of the synthesized AuNPs occurred at a wavelength of 533 nm (Figure 1A). DLS analysis revealed that the mean size of AuNPs was 55 nm, with a polydispersity index (PDI) of 0.22, indicating a homogeneous particle size distribution (Figure 1B). Zeta potential analysis showed that the AuNPs had a zeta potential above -15 mV, suggesting good colloidal stability (Figure 1C). TEM images confirmed that the AuNPs were mainly spherical, with sizes ranging from 20 to 55 nm (Figure 1D). Finally, FTIR was used to analyze the chemical composition and functional groups present on the surface of AuNPs. The FTIR spectrum of PEG-modified AuNPs displayed distinctive absorption bands near 1063 cm^-1^ and 1103 cm^-1^, associated with C-O stretching vibrations, confirming the presence of PEG on the AuNPs' surface. Extra absorption bands within the 1000-1290 cm^-1^ range were linked to C-O stretching vibrations of the PEG polymer. These findings validate the successful coating of AuNPs with PEG, which is crucial for stabilizing the nanoparticles and avoiding aggregation (Figure 1E).

Optimizing the amount of AuNPs in RT-PCR

**Figure 2:**
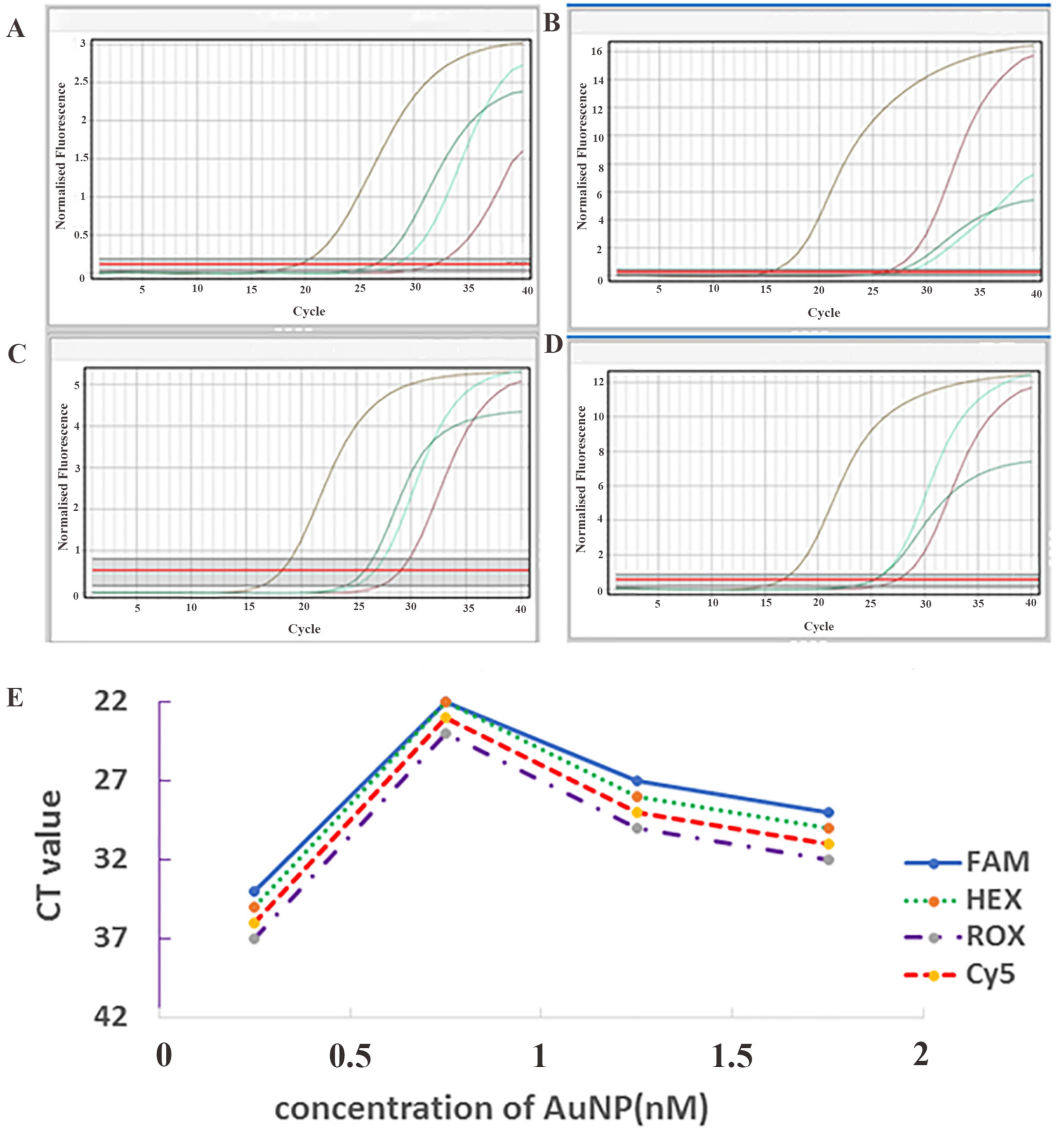
Optimization of AuNP concentration in the master mix A) FAM-labeled HPV 16 probe, B) Cy5-labeled HPV 56 probe, C) HEX-labeled HPV 31 probe, and D) ROX-labeled HPV 18 probe. E) CT values across different AuNP concentrations. The 1 nM concentration showed the lowest CT (fastest amplification), while the 0.5 nM concentration showed the highest CT (slowest amplification). This trend was observed for each of the four HPV types. In A-D, the colors represent: purple=0.5 nM, brown=1 nM, green=1.5 nM, and turquoise blue=2 nM. AuNPs, gold nanoparticles; HPV, human papillomavirus

RT-PCR analysis using different AuNP concentrations showed that 1 nM yielded the lowest CT value, indicating the fastest amplification (Figure 2). In comparison, 0.5 nM showed the highest CT value, suggesting slower amplification. Higher concentrations (1.5 nM and 2 nM) also resulted in increased CT values, indicating reduced amplification efficiency. The optimal AuNP concentration (1 nM) was consistent across all four HPV probe systems (FAM, HEX, ROX, and Cy5), confirming its robustness and adaptability.

Comparison of RT-PCR test results using designed probes and primers with commercially available kits

**Figure 3:**
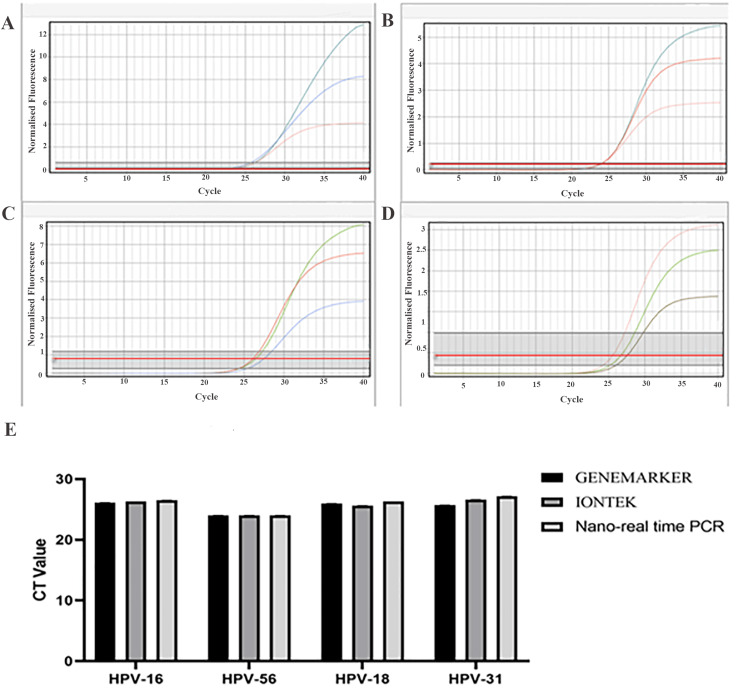
Comparison of nano-RT-PCR results with commercial GENEMARKER and IONTEK kits The results indicated that the kit designed by the present study is comparable to commercial kits. Both commercial and nano-RT-PCR kits detected viral DNA with similar CT values. A) HPV-16: blue = GENEMARKER, green = IONTEK, pink = nano-real-time kit. B) HPV-56: green = GENEMARKER, red = IONTEK, pink = nano-real-time kit. C) HPV-18: green = GENEMARKER, red = IONTEK, blue = nano-real-time kit. D) HPV-31: pink = GENEMARKER, green = IONTEK, brown = nano-real-time kit. E) Comparison of CT values. RT-PCR, nano-real-time polymerase chain reaction; HPV, human papillomavirus

The performance of the nano-RT-PCR method was comparable to 10 commercial kits (GENEMARKER and IONTEK). Viral DNA detection yielded similar CT values across all three systems (Figure 3A-3E), with no statistically significant differences (p>0.05).

Impact of AuNPs on RT-PCR efficiency

**Figure 4:**
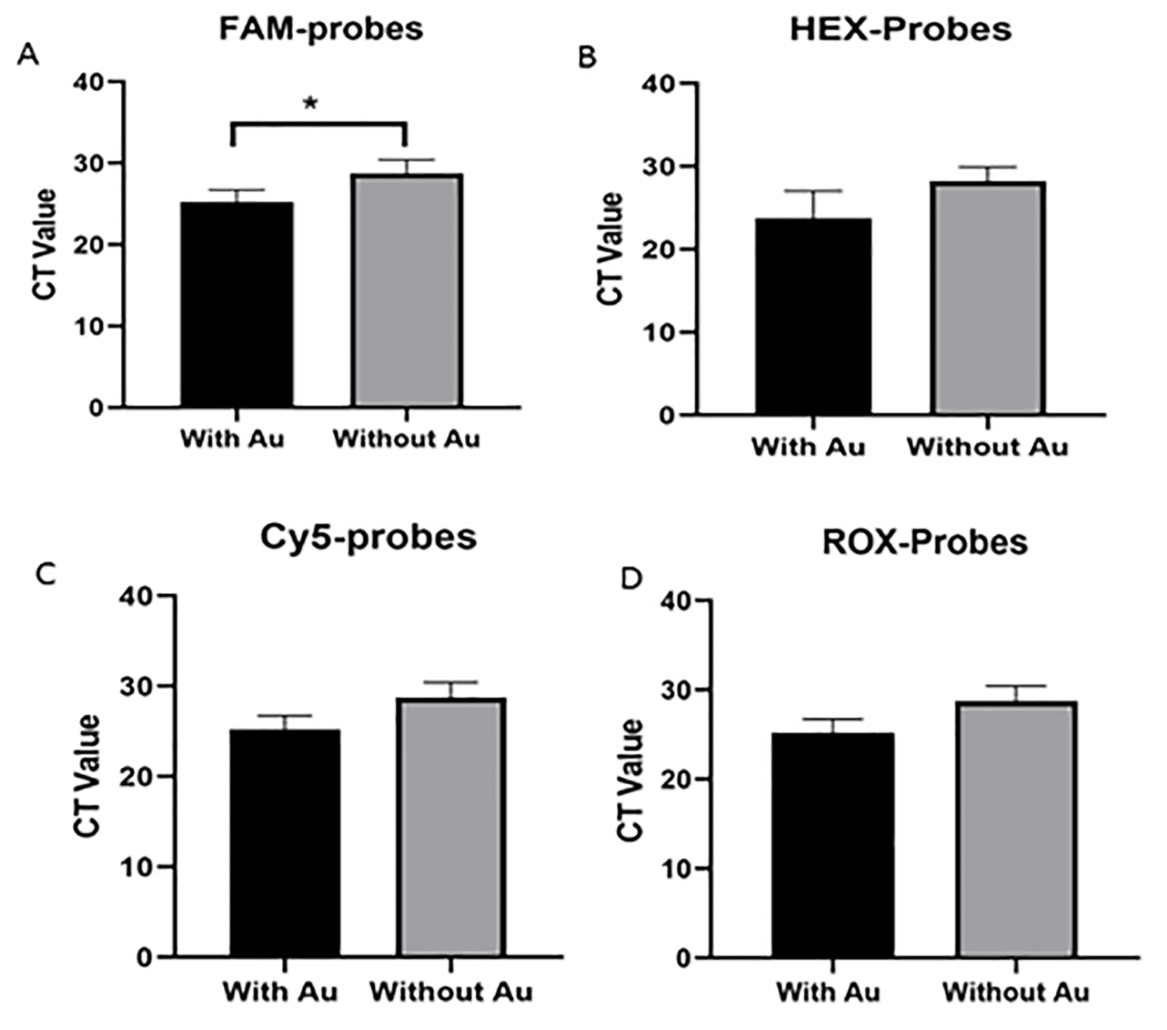
Comparison of RT-PCR results between real-time kits with and without AuNPs for the detection of high-risk HPV types A) FAM probe comparison (with and without Au). B) HEX probe comparison (with and without Au). C) Cy5 probe comparison (with and without Au). D) ROX probe comparison (with and without Au). *The significance level (p<0.05). RT-PCR, nano-real-time polymerase chain reaction; HPV, human papillomavirus; AuNPs, gold nanoparticles

Figure 4 illustrates the results obtained from nano-RT-PCR in comparison with conventional RT-PCR (without AuNPs) for detecting and genotyping HPV strains using dedicated TaqMan probes. The comparison of each HPV type analyzed with the designed kit containing AuNPs and the kit without AuNPs demonstrated a significant difference in CT values. The data suggest that the nano-RT-PCR kit showed lower CT values than the standard RT-PCR kit. This suggests an enhancement in the detection sensitivity of the assay when AuNPs are incorporated. Lower CT values indicate enhanced amplification efficiency and an increased fluorescence signal. Among the various probe sets analyzed, the FAM probe exhibited a statistically significant decrease in CT value with the presence of AuNPs (p<0.05; Figure 4). However, the difference was not statistically significant for the Cy5, HEX, and ROX probes, although a decrease in CY values was observed with the inclusion of AuNPs.

LOD and sensitivity of nano-RT-PCR

**Figure 5:**
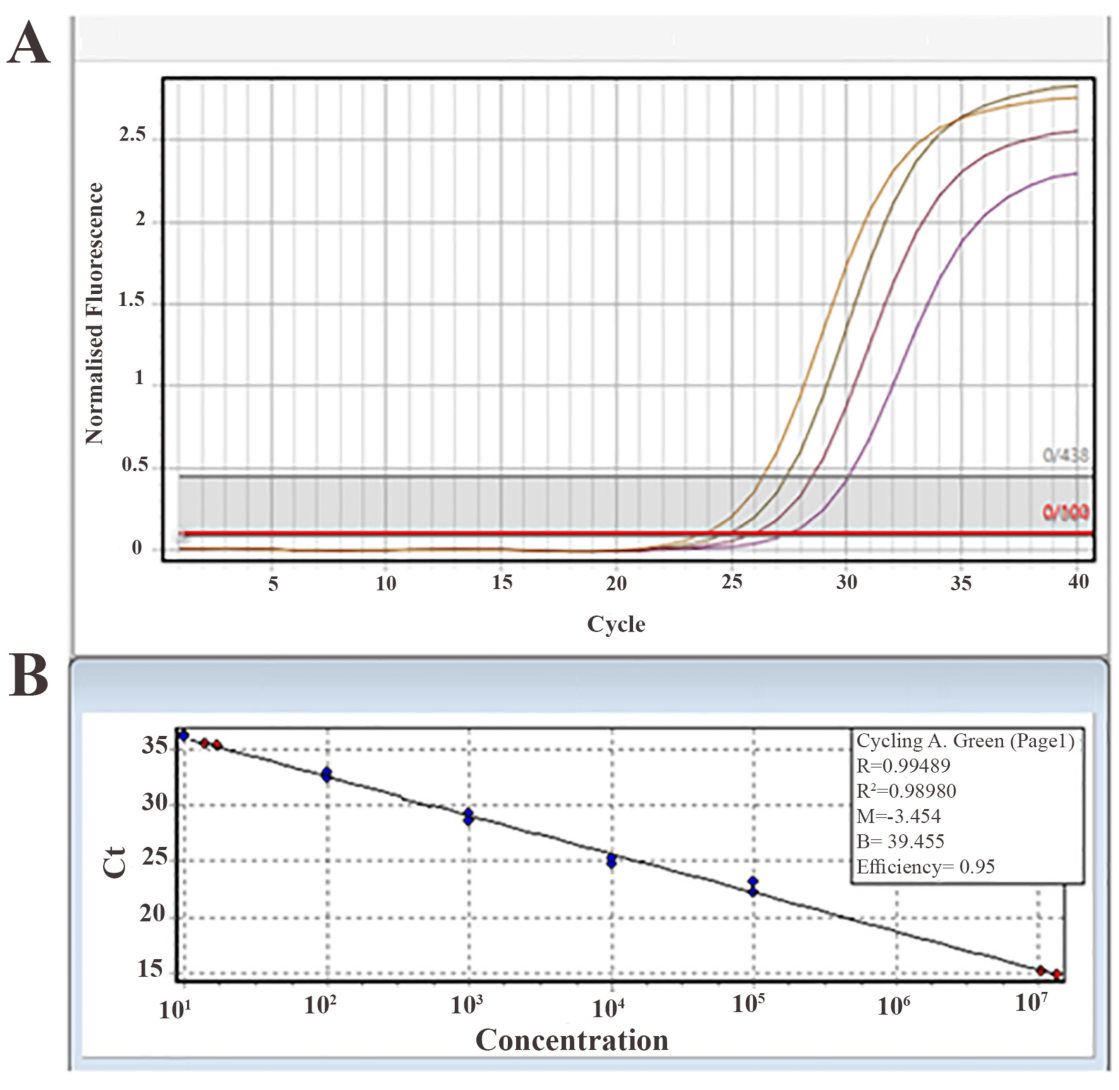
Sensitivity results for the designed kit A) Sensitivity of the nano-RT-PCR kit at different viral DNA concentrations. The colors represent the following viral DNA copy numbers: orange = 10⁶, brown = 10³, crimson = 10², and purple = 10. B) Determination of the LOD for nano-RT-PCR compared with the commercial DynaBio™ HPV 16 & 18 Detection kit. The detection limit for the nano-RT-PCR kit was 16 copies per reaction. RT-PCR, nano-real-time polymerase chain reaction; HPV, human papillomavirus; LOD, limit of detection

To evaluate the sensitivity of the nano-RT-PCR method, serial dilutions of viral HPV 16 DNA at different concentrations were prepared. The findings showed that the minimum detectable concentration using this method was 0.02 ng, demonstrating its high capability in detecting low amounts of target DNA (Figure 5A). To ensure additional validation, samples with 0.02 ng and 1 ng (as a positive control) were analyzed using the commercial DynaBio™ HPV 16 & 18 Detection kit. The findings revealed that the lowest detectable copy number by nano-RT-PCR was 16 copies per reaction (Figure 5B). The assay sensitivity was measured using the standard formula and was determined to be 99%, confirming the high accuracy of this method in detecting positive samples.

Specificity in nano-RT-PCR test

**Figure 6:**
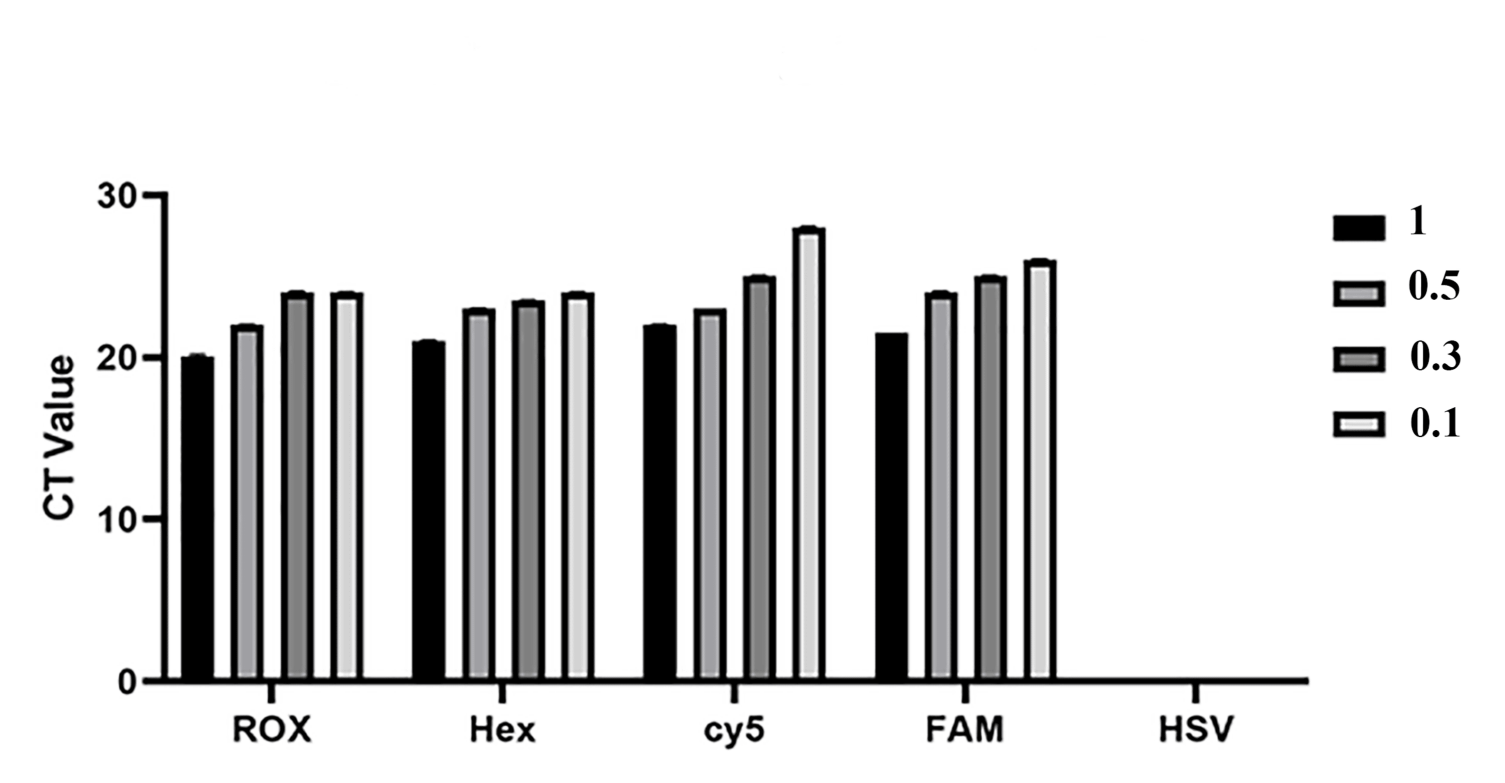
Specificity results for different HPV types compared with HSV No CT was observed at any of the different concentrations of HSV. HPV, human papillomavirus; HSV, herpes simplex virus

Different concentrations of HPV types were tested to evaluate the specificity of the designed assay (Table 3). The results demonstrated that the developed kit, incorporating four distinct reactions with probes labeled with FAM, HEX, ROX, and Cy5 dyes, successfully distinguished HPV from HSV. Notably, no CT value was observed for HSV in any of the tested master mixes, indicating no cross-reactivity (Figure 6).

Moreover, all four types of HPV effectively detected HPV DNA at concentrations as low as 0.3 ng. The assay was validated using four different HPV genotypes, including HPV 16, HPV 18, HPV 31, and HPV 56. All tested HPV types were successfully identified using the master mixes containing AuNPs conjugated to different fluorophores. Based on this calculation, the test's specificity was determined to be 100%. These findings confirmed the high accuracy of the designed assay in distinguishing HPV from HSV, demonstrating its potential for reliable HPV detection in 2026 clinical diagnostics.

## Discussion

This study demonstrates that the controlled incorporation of PEG-modified AuNPs into an RT-PCR system significantly enhances amplification efficiency and analytical sensitivity for high-risk HPV detection. Notably, the optimized nano-RT-PCR assay achieved lower CT values and a detection limit of 16 copies, indicating improved reaction kinetics and signal generation compared with conventional PCR approaches. These performance gains suggest that AuNPs positively influence the PCR microenvironment by promoting more efficient heat transfer, stabilizing enzymatic activity, and facilitating primer-probe hybridization. Together, these findings indicate that nanoparticle-assisted PCR represents a robust and sensitive platform for HPV genotyping, with clear potential to improve molecular diagnostics in clinical settings.

UV-vis and FTIR analyses confirmed the successful synthesis and PEG modification of AuNPs, which is consistent with previous studies [17-22]. The PEG coating improved stability by preventing aggregation and ensuring uniform dispersion. The optimization results showed that the concentration of AuNPs had a significant effect on PCR performance. Among the tested concentrations, 1 nM AuNPs induced the most efficient amplification, as indicated by the lowest CT values. This finding is consistent with the study of Li et al., who reported that the incorporation of AuNPs into PCR mixtures increased the amplification efficiency by improving heat transfer and facilitating more efficient primer-template hybridization [23]. According to their results, AuNPs could enhance local microheating and increase the rate of DNA denaturation and annealing, finally accelerating the PCR process.

However, when the concentration of AuNPs exceeded the optimal level, inhibition of amplification was observed. This observation is consistent with the findings of Wan et al., who showed that AuNPs exert a size- and concentration-dependent inhibitory effect on PCR [24]. They attributed this effect to the interaction between AuNPs and Taq polymerase, where an excessive concentration of nanoparticles leads to nonspecific adsorption of the enzyme on the surface of the nanoparticles, thereby reducing its catalytic activity. This suggests that maintaining an optimal concentration of nanoparticles is crucial to balance the beneficial and inhibitory effects of AuNPs in PCR reactions.

Compared with commercially available PCR kits, the developed nano-RT-PCR method showed comparable performance, confirming the accuracy of the designed primers and probes and the stability of the nano-amplified system. The incorporation of AuNPs into the PCR mixture may enhance enzyme stability, minimize inhibitory effects, and improve the uniformity of reaction kinetics. The reduced CT values observed with the addition of AuNPs indicate an increase in amplification efficiency, which may be attributed to improved hybridization dynamics and more efficient heat distribution during the thermal cycling process.

Furthermore, Lin et al. suggested that AuNPs can enhance PCR efficiency and specificity by simultaneously influencing the electrostatic environment of the reaction [25]. Their work demonstrated that AuNPs facilitate primer-template binding through charge neutralization and steric confinement effects, leading to reduced nonspecific amplification and improved target specificity. These mechanistic insights are consistent with our findings, suggesting that AuNPs play a multifaceted role in promoting thermal homogeneity, stabilizing enzyme activity, and improving probe-target hybridization. Therefore, the data confirm that the controlled incorporation of AuNPs at an optimal concentration can significantly enhance the performance of RT-PCR, making the developed nano-PCR kit a robust and sensitive platform for molecular detection.

Sensitivity evaluation showed a detection limit of 16 copies, which is comparable to or better than previous nanoparticle-enhanced PCR systems [26,27]. This high sensitivity is likely due to the enhanced primer-template interactions and improved fluorescence signaling induced by AuNPs.

Finally, specificity experiments confirmed that the method accurately detects HPV without any cross-reaction from HSV, which is consistent with previous AuNP-based viral detection systems [28,29]. These findings confirm that the developed nano-RT-PCR method offers excellent sensitivity, specificity, and adaptability for HPV detection, confirming its potential for clinical diagnostic applications.

Limitations of the study

Despite the promising results of the developed nano-RT-PCR assay, several limitations should be acknowledged. First, this study was primarily conducted under controlled laboratory conditions using a limited number of clinical samples (n=100). A larger and more diverse sample set would be required to comprehensively validate the assay’s diagnostic accuracy and reproducibility across different populations and clinical settings. Second, although the assay demonstrated high specificity and sensitivity compared with commercial kits, cross-platform reproducibility and inter-laboratory consistency were not evaluated, which may affect generalizability. Third, the study focused on a selected panel of high-risk HPV genotypes. While these include clinically significant strains, the assay’s ability to detect additional or emerging HPV subtypes remains to be established. Expanding the probe and primer design to encompass a broader range of genotypes would further enhance the kit’s applicability.

Additionally, the study did not address potential interference from other biological components present in cervical or anogenital samples, such as blood, mucus, or host DNA, which could influence assay performance in real-world diagnostics. Finally, for specificity analysis, only HSV was tested as a non-target virus. A more thorough specificity panel (e.g., CMV, EBV, or low-risk HPV types) could also be used for a diagnostic assay.

## Conclusions

This study indicated that AuNPs enhance the efficiency, sensitivity, and specificity of TaqMan-based RT-PCR for HPV detection. The optimized 1 nM AuNP concentration improved amplification efficiency and obtained lower CT values across master mixes. The assay matched commercial kits in performance, detected HPV with a 0.02 ng limit, and showed 100% specificity without cross-reactivity. These findings confirm the role of AuNPs in improving PCR accuracy, making this approach a promising tool for reliable HPV diagnostics with broader applications in molecular testing.
